# The practice and clinical implications of tablet splitting in international health

**DOI:** 10.1111/tmi.12309

**Published:** 2014-04-07

**Authors:** Ivo Elliott, Mayfong Mayxay, Sengchanh Yeuichaixong, Sue J Lee, Paul N Newton

**Affiliations:** 1 Lao-Oxford-Mahosot Hospital-Wellcome Trust Research Unit, Microbiology Laboratory, Mahosot HospitalVientiane, Lao PDR; 2 Centre for Tropical Medicine, Nuffield Department of Medicine, Churchill Hospital, University of OxfordOxford, UK; 3 Faculty of Postgraduate Studies, University of Health SciencesVientiane, Lao PDR; 4 Mahidol-Oxford Tropical Medicine Research Unit, Faculty of Tropical Medicine, Mahidol UniversityBangkok, Thailand

**Keywords:** tablet splitting, therapeutic range, Laos, dose, essential medicines

## Abstract

**Objective:**

Tablet splitting is frequently performed to facilitate correct dosing, but the practice and implications in low-income settings have rarely been discussed.

**Methods:**

We selected eight drugs, with narrow therapeutic indices or critical dosages, frequently divided in the Lao PDR (Laos). These were split, by common techniques used in Laos, by four nurses and four laypersons. The mean percentage deviation from the theoretical expected weight and weight loss of divided tablets/capsules were recorded.

**Results:**

Five of eight study drugs failed, on splitting, to meet European Pharmacopoeia recommendations for tablet weight deviation from the expected weight of tablet/capsule halves with 10% deviating by more than 25%. There was a significant difference in splitting accuracy between nurses and laypersons (*P *= 0.027). Coated and unscored tablets were less accurately split than uncoated (*P *= 0.03 and 0.0019 for each half) and scored (0.0001 for both halves) tablets.

**Conclusion:**

These findings have potential clinical implications on treatment outcome and the development of antimicrobial resistance. Investment by drug companies in a wider range of dosage units, particularly for narrow therapeutic index and critical dosage medicines, is strongly recommended.

## Introduction

Tablet splitting is widely practised in many areas of healthcare. In the German primary care setting in 2006, an estimated one quarter of all drugs were split and in a large elderly care home in Canada 35% of all tablets were split (Fischbach *et al*. 2001; Quinzler *et al*. 2006). In low-income settings, such as the Lao PDR (Laos), tablet splitting is commonly performed, but the practice and implications have rarely been discussed.

The primary reason for tablet splitting is to increase dose flexibility, particularly for the elderly, children and those requiring titrating or tapering doses (Fischbach *et al*. 2001; Cohen & Cohen 2002; van Santen *et al*. 2002). Appropriate doses are often not manufactured or are unavailable. Drug costs per unit of active pharmaceutical ingredient (API) frequently decrease with increasing dose or flat charges may exist, independently of dose. Tablet splitting can therefore have an economic incentive, benefitting both the individual patient and the healthcare provider. Estimates of cost saving for splitting statins from innovative pharmaceutical companies are as high as 40–50% (Fawell *et al*. 1999; Duncan *et al*. 2002; Gee *et al*. 2002). This cost saving is, however, limited to relatively few drugs. Finally, a more pragmatic reason for tablet splitting is to aid swallowing.

Splitting tends to be performed by a variety of people including pharmacists, nurses and patients or their relatives. Tablets, particularly those with score lines, are usually split by hand. Those without score lines may require the use of a razor, knife or scissors. Commercially available splitting devices can also be employed where available (e.g. www.medimax.co.uk). Occasionally, capsules are split by emptying the powder and dividing it into equal portions.

The accuracy of tablet splitting has been demonstrated to vary considerably (Rosenberg *et al*. 2002; Teng *et al*. 2002; Polli *et al*. 2003; Verrue *et al*. 2011). Quality standards for uniformity of weight and content of manufactured drugs are outlined in the British (BP), United States (USP) and European Pharmacopeias (EP). These require both the weight and content of the API of whole tablets to be within 85–115% of the intended dose with a relative standard deviation (RSD) less than or equal to 6% (United States Pharmacopeial Convention 1999). BP and USP standards apply only to whole dosage units with no guidance on split tablets. The revised 2008 EP standards for the division of scored tablets allow for no more than 1 in a set of 30 tablets to be outside the 85–115% range (European Pharmacopoeia Supplement 2008). If one tablet falls outside this range, it must fall within 75–125% of the expected mass/content. Although a homogeneous distribution of active drug is generally assumed in unscored tablets, some variation is expected and US Food and Drug Administration (FDA) bioequivalence standards permit variance of ±20%. The FDA, American Medical Society and American Pharmacists Association advise against splitting modified or sustained release, co-formulated, unscored, film-coated, friable or dose-critical tablets (American Pharmacists Association 2003).

The accuracy of tablet splitting is influenced by tablet size, shape, hardness, splitting method and human ability. Small, round or unusual-shaped tablets give rise to the greatest deviations and harder tablets are most likely to fragment or powder, leading to drug loss (McDevitt *et al*. 1998; Polli *et al*. 2003). Dividing tablets into quarters results in even greater ranges of weight differences (Biron *et al*. 1999; Kayumba 2006). Tablets with score lines, especially if deep, tend to split more uniformly (Gupta & Gupta 1988; Kayumba 2006). Hand-split tablets were less uniform than those split by razor, and knife splitting was less accurate than a tablet splitter (Teng *et al*. 2002; Cook *et al*. 2003). Weight loss due to fragmentation and powdering has been reported to vary between minimal (Stimpel *et al*. 1984; McDevitt *et al*. 1998) and 14% for tablet halves (Biron *et al*. 1999).

The implications of tablet splitting are wide ranging, but large deviations from the ideal weight after splitting are likely to be especially important (McDevitt *et al*. 1998; Rosenberg *et al*. 2002; Teng *et al*. 2002; Polli *et al*. 2003). This may result in incorrect dosing affecting clinical outcome, especially for medicines with narrow therapeutic indices. Under dosing may be an important factor in the development of resistance in diseases such as malaria (White *et al*. 2009a).

We investigated the practice of tablet splitting in the low-income setting of Laos. We performed a study to replicate the usual circumstances and methods by which tablets are split and report the frequency with which split study drugs failed to meet EP guidelines on weight (dose) uniformity and identify factors that may influence the accuracy of tablet splitting.

## Methods

### Participants

Eight Lao volunteers were recruited to perform the tablet splitting at Mahosot Hospital, Vientiane. Four paediatric nurses at Mahosot Hospital and four laypersons (representing patients and relatives) with no healthcare-related experience were selected. These are the two major groups responsible for tablet splitting in Laos (pharmacists are less frequently involved).

### Study drugs

Eight medicines frequently split in Laos, with narrow therapeutic indices or critical dosages, were selected (Table1 and Table S1). Participants were requested to divide all drugs into halves with the exception of phenobarbitone, where one set was divided into halves and another into thirds, as is commonly required in paediatrics. For each study drug, 80 tablets (160 for phenobarbitone) were purchased from a local pharmacy, of the same dose, manufacturer and lot number per medicine. Doxycycline capsules are also frequently divided in Laos for paediatric dosing and were included in the eight study drugs. All splitting was performed before drug expiry date.

**Table 1:** Characteristics of eight commonly split tablets in Laos used in the experiments

Drug	Dose and formulation	Shape	Flat?	Score line?	Coated?	Split into?	Whole tablet weight (g) (SD)
Chloroquine	250 mg T	Round	No	No	Yes	2	0.804 (0.019)
Doxycycline	100 mg C	Oblong	N/A	N/A	N/A	2	0.328 (0.011)
Ofloxacin	200 mg T	Oblong	Yes	Yes	No	2	0.466 (0.011)
Enalapril	20 mg T	Hexagonal	No	Yes	No	2	0.201 (0.002)
Atenolol	100 mg T	Round	No	Yes	Yes	2	0.434 (0.005)
Digoxin	0.25 mg T	Round	Yes	Yes	No	2	0.155 (0.006)
Glibenclamide	5 mg T	Oblong	Yes	Yes	No	2	0.160 (0.001)
Phenobarbitone	60 mg T	Round	No	No	No	2 or 3	0.133 (0.001)

T, tablet; C, capsule; SD, standard deviation.

Manufacturer and excipients are listed in supplementary material.

### Tablet splitting

Participants worked separately and were provided with 10 tablets or capsules of each study drug (total 90 tablets or capsules). They were asked to divide these, using their preferred method, into halves (both halves and thirds for phenobarbitone) and return the pieces or powder into separately labelled zipped plastic bags. Halves are referred to as A and B and thirds as A, B and C.

### Weighing

Each individual whole tablet or capsule was weighed the day before splitting using a Sartorius BL210S (Göttingen, Germany) analytical balance. Weight was recorded to 0.001 g, and whole, half and tablet thirds were weighed using the same machine. Zipped bags containing doxycycline powder were weighed before and after washing out the powder with tap water (followed by thorough drying) as well as the capsule itself to accurately determine the weight of each half of powder. The same individual, blinded to the initial whole tablet weight, weighed the divided tablets/capsules. Interobserver variability was assessed using 50 randomly selected pairs of tablets and two observers.

### Data analysis

Mean whole tablet weight [range and standard deviation (SD)] was calculated for each study drug. The median percentage deviation [interquartile range (IQR)] from the theoretical expected weight of split formulations and the maximum percentage deviation were calculated. A Wilcoxon matched-pairs signed-ranks test was used to compare the weight of each study drug half (or third) and its theoretical weight. Median weight loss, IQR, and maximum weight loss for each study drug were determined. The frequencies (%) of split study drugs falling outside the USP/EP recommended ranges of >85% and <115%, >75% and <125% and >6% relative SDs are reported.

The percentage deviation from the expected weight and the percentage weight loss were compared between coated and uncoated tablets, tablets with and without a scoreline and nurses and laypersons using a Mann–Whitney *U* test. Methods used to split tablets were analysed using the Kruskal–Wallis test. Fifty randomly selected pairs of half tablets from one randomly selected drug were re-weighed to check for interobserver variability using the Bland–Altman method (Bland and Altman 1995).

## Results

Eight drugs (with phenobarbitone used twice) were included in the study with a broad range of tablet characteristics of size, shape, coating and presence of a scoreline plus one powder-filled capsule (doxycycline) formulation. The percentage RSD of whole tablet weight ranged from 0.63% for glibenclamide to 2.63% for chloroquine, well within the recommended maximum of 6% (Table1).

By Bland–Altman analysis for interobserver variability, the mean differences were normally distributed and no significant difference was seen between the first weights and re-weighing (mean difference 0.000856 (95% CI 0.000478–0.00219) g for half A and 0.000578 (0.000438–0.000718) g for half B.

A statistically significant difference (*P *< 0.01) was seen for median percentage deviation from theoretical for split drugs for four of nine sets of tablets/capsules (Table2). Digoxin tablets (*P *= 0.0006) and the unscored and coated chloroquine (*P *= 0.01) were both inaccurately split. Phenobarbitone was the most inaccurately split (*P *< 0.0001) when divided into 3 (not when divided into 2). Dividing the powder content of doxycycline capsules also resulted in significant deviation from expected weight (*P *< 0.01). Median weight loss of tablets for each study drug ranged from 0.22% to 3.75% (Table2). Maximum weight loss varied greatly from 1.85% (glibenclamide) to 23.48% (phenobarbitone halves).

**Table 2:** Median deviation from theoretical weight for each divided study drug and median and maximum weight loss

Drug	Median% deviation from theoretical of half A (IQR)	P*	Median% deviation from theoretical of half B (IQR)	P*	Max% deviation from theoretical of half A or B	Median% weight loss (IQR)
Chloroquine	6.63 (3.00–13.96)	0.8686	8.31 (3.80–15.66)	0.0101	48.97	1.24 (0.74–2.25)
Doxycycline	11.76 (4.57–16.13)	0.0035	7.24 (3.87–15.17)	0.0142	43.97	3.75 (2.02–5.83)
Ofloxacin	2.39 (1.03–4.88)	0.9007	2.27 (1.27–5.66)	0.2286	18.71	0.22 (0.21–0.43)
Enalapril	5.03 (2.46–7.49)	0.2804	4.95 (2.00–8.37)	0.2249	20.20	0.50 (0.49–1.00)
Atenolol	6.59 (2.41–11.88)	0.9840	7.30 (2.31–13.97)	0.0556	45.37	1.14 (0.69–1.45)
Digoxin	7.19 (3.80–14.38)	0.4867	8.92 (3.59–16.03)	0.0006	56.69	1.95 (1.25–2.84)
Glibenclamide	4.67 (2.51–4.97)	0.1053	4.38 (2.50–6.69)	0.9655	30.00	0.62 (0.00–0.63)
Phenobarbitone	9.43 (3.77–19.47)	0.5250	9.85 (3.03–19.77)	0.1090	80.45	1.50 (0.75–2.79)
Phenobarbitone†	15.47 (8.27–23.16)†	0.001			100	2.27 (1.50–3.99)
	12.83 (5.32–27.84)†	<0.0001				
	16.54 (9.09–23.88)†	<0.0001				

All drugs were divided into halves, labelled half A and half B, or thirds, labelled A, B and C.

* Wilcoxon matched-pairs signed-ranks test. *P* value* *= actual weight of divided halves (or thirds) compared with the theoretical expected weight (i.e. whole tablet weight divided by 2 or 3).

† Split into thirds.

Table3 shows the frequency of tablet halves (or thirds) falling outside the USP/EP recommendations. In total, 336 (25%) of the divided tablets were outside 85–115% of the theoretical weight and 140 (10%) deviated by more than 25% from the theoretical weight. Six of nine sets of tablets failed to meet the EP requirements following splitting, although one of these (glibenclamide) only failed on the basis of a single tablet divided inaccurately (27.5 and 30% deviation from theoretical for each half). When phenobarbitone was divided into 3, half the pieces deviated by >50% from the mean theoretical weight.

**Table 3:** Frequencies of tablet halves or thirds deviating by more than 15% or 25% and study drugs with a >6% RSD

Drug	No. of halves (or thirds) deviating by >15% (%)	No. of halves (or thirds) deviating by >25% (%)	>6% RSD
Chloroquine	40/160 (25)	19/160 (12)	Yes
Doxycycline	44/160 (0)	18/160 (0)	Yes
Ofloxacin	4/160 (2.5)	0/160 (0)	No
Enalapril	7/160 (4.4)	0/160 (0)	No
Atenolol	29/160 (18)	10/160 (6.3)	Yes
Digoxin	39/160 (24)	19/160 (12)	Yes
Glibenclamide	1/160 (0.6)	1/160 (0.6)	No
Phenobarbitone	53/160 (33)	21/160 (13)	Yes
Phenobarbitone*	119/240 (50)	52/240 (22)	Yes
Total	336 (25)	140 (10)	

* Split into thirds.

Comparing nurses with laypersons, splitting by nurses resulted in less deviation from theoretical weight of tablet halves (*P *= 0.0273), but not for weight loss. The greatest difference was seen for the division of doxycycline capsules (*P *= 0.0009). There was a significantly greater deviation from theoretical weight and weight loss for unscored (chloroquine and phenobarbitone) tablets than scored tablets (*P *= 0.0001 for both half weights and weight loss). A similar result was seen for coated (chloroquine and atenolol) versus uncoated tablets (*P *= 0.030 and 0.0019 for each half). Weight loss was also significantly higher for coated tablets (*P *= 0.0001).

Significant differences were seen between the different methods used to divide the tablets or capsules for both weight deviation from theoretical weight and weight loss (all *P* ≤ 0.0001). The greatest differences were seen when dividing the powder in doxycycline capsules [median weight deviation of half A 11.74% (4.57–16.13) and half B 7.24% (3.87–15.17)], followed by knife [half A* *= 11.03% (2.38–22.02) and half B* *= 9.43% (3.76–21.80)] and scissors [half A* *= 6.92% (3.14–13.64) and half B* *= 7.58% (3.03–13.73)]. Dividing tablets by hand was most accurate [half A* *= 3.86% (1.88–6.80) and half B* *= 3.28% (1.69–6.25)].

## Discussion

Tablet splitting is widely practised throughout the world, but has rarely been examined in low-/middle-income countries. In this study in Laos, six of the nine sets of split tablets/capsules failed to meet EP guidelines on weight uniformity of divided fragments, with 25% deviating by >15% and 10% by >25%. Previous studies report seven of 22 split drugs (Rosenberg *et al*. 2002), eight of 11 (Teng *et al*. 2002), four of 12 (Polli *et al*. 2003) and seven of eight (Verrue *et al*. 2011) failing to meet USP/EP guidelines. These findings are broadly similar to our own, despite the use of different tablets and methods of splitting.

Three study drugs, glibenclamide, ofloxacin and enalapril, were accurately divided and suffered the least weight loss (median <0.62%), and all participants reported these to be simple to divide either by hand or using scissors. All three formulations were scored and lacked a coating. Both these factors were significantly associated with more accurate tablet division. Atenolol and digoxin halves failed to meet EP guidelines. Digoxin had a statistically significant difference between weight of halves and theoretical weight; however, atenolol did not quite reach statistical significance (*P *= 0.0556). This was despite the presence of a scoreline in both tablets. Atenolol was coated, rounded and hard making it very difficult to split by hand and easy to fragment into multiple pieces when using knife or scissors. Digoxin tablets were very small, soft and crumbled easily when divided, reflecting a marked median weight loss of drug (1.95%). Phenobarbitone was a very small, round and hard tablet. Division into two pieces resulted in failure to meet the EP guidelines, but as for atenolol, this did not reach statistical significance for the weights of the halves compared with the theoretical weight (*P *= 0.109). Division into three pieces proved the most difficult with very large differences between the weight of thirds and theoretical weight (*P *< 0.0001), median weight loss of 2.27% and as much as a 100% deviation from theoretical weight. Dividing the powder from doxycycline capsules in half also proved difficult and resulted in significant differences in weight and the greatest median weight loss (3.75%). There was a statistically significant difference in results seen between nurses and laypersons, with nurses performing more accurate division. We selected paediatric nurses working at Mahosot Hospital, who regularly divided drugs as part of their practice. This experience is likely to have resulted in greater accuracy of division.

Median tablet/capsule weight losses due to splitting in this study were 0.22–3.75%, consistent with the few other studies to report this (Gupta & Gupta 1988; Teng *et al*. 2002). In our study, a maximum weight loss of 23.5% for a phenobarbitone tablet was seen. One study reported a maximum of 27% weight loss when dividing oral anticoagulants into quarters (Biron *et al*. 1999).

The clinical impact of these often-large variations in weight between fragments has been investigated primarily for drugs with wide therapeutic indices. The impact of RSDs of more than 10% of halved statins had no clinical impact on patient LDL cholesterol or total cholesterol (Duncan *et al*. 2002; Gee *et al*. 2002), and similar variations of weight of lisinopril fragments did not impact blood pressure over a prolonged follow-up period (Rindone 2000). One study examined content uniformity of the narrow therapeutic index levothyroxine after splitting and suggested that sub- or super potency could prove clinically detrimental; however, no clinical studies were performed (Shah *et al*. 2010).

No specific recommendations were provided by the manufacturers of any of the study drugs, scored or unscored, specifically advising on whether tablet or capsule splitting could be performed. The uniformity of distribution of API in the study drugs was also not recorded. Scored tablets facilitate splitting, and the importance of manufacturing quality assurance standards for API distribution in whole tablets has recently been highlighted (Anonymous 2014). In the absence of explicit documentation by the manufacturer allowing splitting of unscored or coated tablets, we recommend education of healthcare staff and patients against dividing these tablets. Best practices guidelines for tablet splitting are available (US Food & Drug Administration). However, where alternative dosage units do not exist, manufacture of these is thus urgently needed.

Inadequate antimalarial treatment doses, particularly in patients with hyperparasitaemia, may be an important source of *de novo* resistance and recrudescence (Simpson *et al*. 2000; White *et al*. 2009b). One study performed in Africa found that 13% of quinine sulphate tablets deviated in weight by more than 35% from the theoretical (Kayumba 2006). Our study demonstrated difficulty in accurately splitting chloroquine tablets, which may be an important factor in developing drug resistance. Subtherapeutic doxycycline dosing in severe malaria may also be a factor in emerging resistance for partner artemisinin drugs (Newton *et al*. 2005; Dondorp *et al*. 2009) and could result in resistance developing in bacteria such the rickettsiae for which doxycycline is frequently used in Laos and elsewhere in Asia. Tablet splitting is contraindicated for co-formulated drugs including the widely used amoxicillin/clavulanic acid (American Pharmacists Association 2003; Anonymous 2014). Although not investigated in this study, division of this antibiotic may also act as a driver for resistance.

Our findings are likely to underestimate the effect of tablet splitting. Divided tablets may fragment further when kept in a container after splitting, hygroscopic absorption from high humidity and the transfer of skin oils onto the tablets may have overestimated fragment weights. We were unable to determine the uniformity of distribution of API in whole tablets. Uneven distribution of API in divided tablets may lead to even greater risks for the emergence of antimicrobial resistance. We did not perform a direct comparison of splitting techniques to determine an optimal method, but division by hand appeared to be most accurate, followed by scissors or knife. Least accurate was the division of powdered drug from capsules. The accuracy of splitting by hand may reflect the choice available to participants, as tablets that were easy to divide, for example ofloxacin was divided so by hand, but the most difficult to split, for example atenolol, could only be divided using scissors or a knife. Verrue *et al*. (2011) showed that a splitting device was significantly more accurate than division with scissors or by hand; however, these devices are expensive and unavailable in low-income settings. An additional limitation to this study was that participants split a large number of tablets in a period of approximately 90 min. This does not reflect usual practice and may have influenced accuracy.

Further analysis should be performed to better understand the pharmacokinetic–pharmacodynamic consequences of tablet splitting for particular pathogens/disease states. That division of uncoated tablets with scorelines resulted in the most accurate tablet division suggests that pharmaceutical manufacturers of medicines that are commonly split should consider such lines, if technically feasible, based on pharmaceutical specifications including the proof of uniform distribution of API. These data suggest that some key medicines such as doxycycline capsules, chloroquine and digoxin tablets should not be split and that phenobarbitone tablets should not be split, especially into thirds.

## Conclusion

This study highlights the widespread practice and inaccuracy of tablet and capsule splitting of medicines. There is clear evidence that tablet design, with the lack of a coating and presence of a scoreline, allows significantly more accurate tablet splitting. The potential clinical implications of this are far reaching and may have a significant impact on successful outcome and the development of antimicrobial resistance. Vulnerable groups including paediatric and elderly patients are most at risk of the consequences of inaccurate tablet division. Investment by drug companies in the production of a wider range of dosage units or tablets better designed for splitting and better product information on the suitability of tablets for splitting, particularly for narrow therapeutic or critical dosage drugs, is strongly recommended.

## Supporting Information

Table S1 Study drug tablet/capsule excipients.
