# Supplementary material for: The practice and clinical implications of tablet splitting in international health
Source: Trop Med Int Health. 2014 Apr 7;19(7):754–60. doi: 10.1111/tmi.12309 (PMC4285309; doi:10.1111/tmi.12309)
Supplement: Table S1 — Study drug tablet/capsule excipients. [file tmi0019-0754-SD1.docx]

**Supplementary Table 1.** Study drug tablet/capsule excipients

| Drug | Excipients |
| --- | --- |
| Chloroquine * | Not available |
| Doxycycline * | Microcrystalline cellulose (Avicel 102), corn starch, lactose monohydrate, sodium lauryl sulfate, anhydrous colloidal silicon dioxide (Aerosil 200), magnesium stearate |
| Ofloxacin * | Lactose monohydrate, corn starch, microcrystalline cellulose (Avicel ph 101), povidone K30, sodium starch glycolate, talc, magnesium stearate, Pharmacoat 615, polyethylene glycol 6000, titanium dioxide |
| Enalapril # | Lactose monohydrate, microcrystalline cellulose (Avicel ph 101), sodium starch glycolate, maleic acid, magnesium stearate |
| Atenolol # | Corn starch, dibasic calcium phosphate dihydrate, sodium starch glycolate, carboxymethylcellulose calcium, magnesium stearate, purified water |
| Digoxin ^ | Lactose, corn starch, talc, tapioca starch, Aerosil, magnesium stearate |
| Glibenclamide § | Magnesium stearate, unspecified compression material |
| Phenobarbitone § | Lactose, tapioca starch, microcrystalline cellulose (Avicel ph 101), Povidone K25, corn starch |

** Manufactured by Codupha-Lao Pharmaceutical Factory, Dongkhamxang Rd, Xiengda Village, Vientiane, Lao PDR*

# *Manufactured by Berlin Pharmaceutical Industry Co. Ltd., Latkrabang, Bangkok 10520, Thailand*

*^ Manufactured by Pharmaceutical Factory No 2, Sokpaluang Rd, Vientiane, Lao PDR*

§ *Manufactured by Government Pharmaceutical Organization, 75/1 Rama VI Rd, Ratchatewi, Bangkok 10400, Thailand*
